# Willingness to Pay for Food Labelling Schemes in Vietnam: A Choice Experiment on Water Spinach

**DOI:** 10.3390/foods11050722

**Published:** 2022-02-28

**Authors:** Duc Tran, Ieben Broeckhoven, Yung Hung, Nguyen Hoang Diem My, Hans De Steur, Wim Verbeke

**Affiliations:** 1 Department of Agricultural Economics, Ghent University, 9000 Ghent, Belgium; ieben.broeckhoven@kuleuven.be (I.B.); yung.hung@ugent.be (Y.H.); hans.desteur@ugent.be (H.D.S.); wim.verbeke@ugent.be (W.V.); 2 Department of Earth and Environmental Sciences, KU Leuven, 3000 Leuven, Belgium; 3 Faculty of Economics and Development Studies, University of Economics, Hue University, Hue City 52000, Vietnam; nhdmy@hueuni.edu.vn

**Keywords:** willingness to pay, labelling, food quality and safety, consumer

## Abstract

The growing concern for food safety and quality motivates governments and private sectors to improve consumers’ confidence in food systems, such as through adopting certifications and traceability systems. The recent emergence of diverse food labelling schemes and the turbulence in food systems in emerging countries have sparked questions about consumers’ valuation of such labels. Nonetheless, little is known on how the familiarity with, trust in and knowledge of these food labelling schemes affect consumers’ willingness to pay for labelling schemes in emerging market contexts. This study aims to address these literature gaps by investigating consumers’ valuation of existing certifications, branding and traceability labelling schemes in Vietnam. A face-to-face survey was conducted, including a discrete choice experiment on water spinach in Ho Chi Minh City, Vietnam. The findings indicated that Vietnamese consumers are generally willing to pay price premia for food labelling schemes, such as VietGAP certification, EU and USDA organic certifications, private branding and traceable Quick Response (QR) coding. While familiarity and understanding had no significant impact on Vietnamese consumers’ valuation, trust was found to be a critical factor shaping willingness to pay for products bearing VietGAP label. Policy implications and marketing strategies for organic certifications and traceability schemes in Vietnam are discussed.

## 1. Introduction

Food safety is a global problem as almost one in ten people worldwide fall sick due to contaminated food, and 420,000 die every year due to foodborne diseases [1]; however, consumers’ awareness of food safety remains modest worldwide [2,3]. Recent food frauds, such as melamine in infant formula milk in China [4], horsemeat in beef burgers in Ireland [5] and food safety risks such as mad cow disease [6], have increasingly captured public attention [7]. Subsequently, consumers have lost confidence in food safety in developed and developing countries [8]; however, food safety problems are especially severe in developing countries [9]. The World Bank estimated that low- and middle-income countries account for 53% of all illnesses and 75% of deaths related to foodborne diseases worldwide, while these countries represent only 41% of the world population [10].

In emerging countries, food safety and quality have gradually improved due to increased agricultural export and growing domestic demands [11]. Agricultural export generally implies applying more stringent standards regarding food safety and quality for imported products than national standards in emerging countries [12]. Consequently, exporters in emerging countries need to upgrade their production standards to reach lucrative markets in developed countries. Further, as emerging countries’ income levels are rising, domestic demand for food quality and safety tends to increase [9].

Consumers cannot ascertain many food safety and quality attributes before or after purchase [13]; therefore, food producers need to communicate food quality and safety attributes through, e.g., labelling schemes such as written information, logos and embedded information in scannable codes. Previous studies showed that consumers expressed a positive attitude, preference and willingness to pay for products indicating food quality and safety attributes on their packages [14,15,16].

The emergence and development of various food labelling schemes are giving rise to a multitude of questions on how consumers value these schemes. Many studies have been conducted in developed countries [17,18], but similar studies remain scarce in emerging countries [9], with China being the exception [19,20]. Nevertheless, studies on consumer valuation of food labelling schemes in emerging countries are critical as developed countries’ findings cannot be extrapolated. Furthermore, the situation in emerging countries differs from developed countries because (i) enforcement and regulation of food safety and quality laws, which can influence consumers’ trust in the food systems, are less strict [21]; (ii) traceability and certification schemes are relatively new to consumers. Thus, consumers might not be well-informed nor familiar with such schemes and related products [22,23].

Enhancing the understanding of consumers’ valuation of food labelling schemes can benefit all food chain actors, allowing policymakers to develop more effective regulations tailored to consumers’ needs. Simultaneously, this improved understanding of consumer valuation will facilitate agricultural producers and retailers to evaluate the cost and benefit of implementing new certification schemes. Furthermore, this could also enable food chain actors to examine the potential of traceability as a value addition method for agricultural products.

As an emerging country, Vietnam has also experienced severe food safety issues. During 2000–2019, more than 3000 outbreaks have been recorded, with almost 100,000 cases of food poisoning, causing almost 800 deaths in Vietnam [24]. Even more so, these figures might be underestimated as the World Bank announced that food poisoning outbreaks in Vietnam are frequently underreported [25]. As a result, the Vietnamese government and food producers have taken several measures, namely (1) adopting certification schemes, (2) promoting brand images and (3) implementing traceability systems.

Several certification schemes have recently been developed and adopted in Vietnam [22]. The most popular certification is VietGAP, a voluntary certification scheme adapted from GlobalGAP (Global Good Agriculture Production), stipulating agricultural production standards in Vietnam. While the Vietnamese government has issued general standards for organic production, processing and labelling, implementation is lacking [26]. By contrast, a non-governmental organic certification, Participatory Guarantee System (PGS), has been established and expanded; however, PGS is only implemented in a few provinces at a modest scale [26].

Food brands have focused on brand images regarding food quality and safety as recent food scandals have concerned consumers in Vietnam [8]. Moreover, food corporations frequently display their brand logos alongside certification logos on food packages to gain trust from consumers [12].

The provision of detailed traceability information to consumers has recently been stimulated due to the Internet and advancements in Information and Communication Technology (ICT) in Vietnam [27]. As a result, Vietnamese consumers can scan Quick Response (QR) codes on some food packages to retrieve traceability information.

## 2. Literature Review on Consumers’ Preferences for Food Labelling Schemes in Vietnam

Many previous studies on consumers’ assessment of organic food either did not clarify or examine a specific organic certification in Vietnam [28,29]. In these studies, the “organic” attribute was often briefly described as “not using genetically modified organisms and synthetical chemicals in cultivation”. Only one study, by using a choice experiment, assessed Vietnamese consumers’ willingness to pay for PGS organic certification [21] as this certification is prevalent in Hanoi and the north of Vietnam [30]. Even though produce with European (EU) and United States Department of Agriculture (USDA) organic certifications are increasingly consumed in Vietnam, no Vietnamese consumer studies for these certifications were conducted to the best of our knowledge. As such, there is a need for comparing national and international certification schemes to illustrate the competitiveness of Vietnam’s market.

Vietnamese consumers’ perception and valuation of food products are prominently affected by brand trust [8]; however, studies eliciting consumers’ valuation for food branding remain scarce. Wongprawmas and Canavari [12] examined consumers’ valuation for certification and branding in Bangkok, Thailand, but called for further research that separates the impact of certification and branding attributes in their choice experiment.

Dang et al. [31] examined consumers’ valuation for traceability and certification schemes of water spinach in Vietnam, but they did not elicit the willingness to pay for specific schemes. Previous studies in Vietnam presented traceability attributes as stated information on packages in a choice experiment, but not as embedded information, such as QR codes or bar codes [28,29,31].

Effects of consumers’ characteristics on the willingness to pay for food labelling schemes in Vietnam have remained largely unexplored [21,28,29]. Nevertheless, consumer characteristics such as familiarity, trust and knowledge are deemed to be important determinants of consumers’ WTP for food safety labelling attributes in Vietnam. Since the examined certification schemes have been launched in Vietnam in different periods, it is of interest to investigate the impact of consumers’ familiarity on consumer choice in relation to these certifications [22]. Moreover, consumers’ lack of knowledge regarding organic farming has been considered an essential factor that hinders organic market growth [32,33]. Furthermore, brand trust plays a critical role in building consumers’ trust in safe vegetables in Vietnam [8]. Consumers often distrust the credibility of current safety certifications due to the recent food safety scandals [31], which might profoundly affect consumers’ valuation.

This study aims to address the aforementioned literature gaps through a choice experiment for water spinach in Ho Chi Minh City, Vietnam, to elicit willingness to pay for specific food labelling schemes, including certifications (VietGAP, EU and USDA organic certifications), a private brand logo and a traceable QR code. Water spinach was chosen as a product of interest as it is widely consumed and represents a part of traditional cuisine in Vietnam [34]. Additionally, a survey was administrated in Ho Chi Minh City, where most examined labelling schemes recently emerged. The choice experiment data were further analysed by generalised multinomial logit models to assess consumer preferences and scale heterogeneity. To understand consumers’ willingness to pay better, individual specific characteristics such as income, education levels and consumers’ characteristics, such as familiarity, trust, and knowledge of labelling schemes, were included.

## 3. Materials and Methods

### 3.1. Data Collection and Survey

A pilot survey (n = 25) was conducted in December 2019 to verify the questionnaire’s clarity and modify the choice experiment design. After revisions, the survey was finalised and administered in-person by trained interviewers at food stores in Ho Chi Minh City, Vietnam, in February 2020. The survey (n = 300) was conducted at different times during the week to cover a diverse set of consumers. Interviewers stayed near the fresh fruits and vegetable shelves and asked shop visitors to participate voluntarily. Shopping vouchers of VND 30,000 (VND = Vietnamese dong; 1 EUR ≈ VND 25,300 (In February 2020)) in value were given to the participants who completed the survey. The survey consisted of three sections. The Section 1 pertained to familiarity, trust and knowledge of VietGAP, organic certifications and Coop-Food logos. If there is no further clarification, the term “organic” (logos) refers to EU and USDA organic certification (logos) in this paper. Purchase habits and intentions for VietGAP and organic certified water spinach were also incorporated. The descriptions of mentioned questions and variables in Section 1 can be found in Appendix A. The choice experiment is shown in the Section 2, as described in Section 3.2. The Section 3 consisted of questions relating to respondents’ demographic and socio-economic characteristics. Before the survey, all participants were informed that their participation was entirely voluntary and that their data would be pseudo-anonymised and protected in line with the General Data Protection Regulation (EU) 2016/679. As twenty-five respondents chose not to disclose their income and education, their data were excluded from subsequent analyses and modelling, thus yielding a valid sample of 275 participants.

### 3.2. Discrete Choice Experiment

A Discrete Choice Experiment (DCE) was used to elicit consumer valuation of certification, traceability and branding. As non-hypothetical methods such as auction experiments are expensive, hypothetical methods such as DCE are commonly chosen [35]. A DCE is based on choice modelling that assumes (1) a good possesses a bundle of attributes that contribute to consumers’ utility [36] and (2) consumers purchase a good or service to maximise their utility [37]. In the DCE setting, respondents are shown choice sets with multiple alternatives. Each alternative has a set of attributes. Each attribute, in turn, has different levels, which vary among alternatives. By observing the trade-off decisions when choosing several similar goods, one can estimate the utility contributed by each attribute of a good and derive a hypothetical willingness to pay for each attribute.

**Figure 1:**
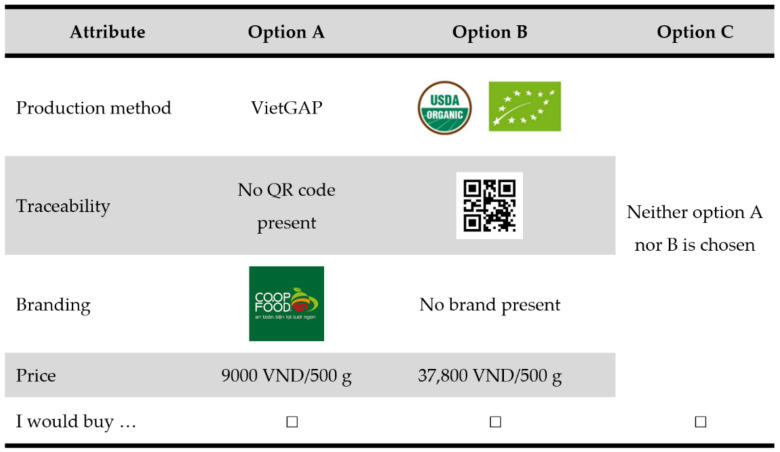
Example of a choice set.

Each choice set consisted of three choice options: option A, option B and an opt-out. The first two choice options were presented as a package of water spinach (500 g), including four product attributes, namely (1) production method, (2) branding, (3) traceability and (4) price (Figure 1). Other attributes were assumed to be ceteris paribus, which was explained to respondents before completing the choice experiment.

Each attribute and its attribute levels were chosen to represent the Vietnamese market context for vegetables. The production method consisted of three levels, namely VietGAP certification, EU and USDA organic certifications, and conventional method. As no unified VietGAP logo is available [22], the word “VietGAP” was used to illustrate the VietGAP certification in the choice sets (Figure 1). EU and USDA organic logos were chosen since products with these attributes are increasingly consumed by Vietnamese consumers [38]. In this study, the EU and USDA organic logos were placed alongside each other within the same choice option, because they have equivalent use for marketed vegetables [39] and usually appear together on the label/food packages in Vietnamese markets. The conventional production method refers to water spinach produced without a certification scheme. The Coop-Food brand logo and QR code illustrated the branding and traceability attributes, respectively, and were included due to their popularity in the Vietnamese market [31,40]. The price attribute consisted of six levels, ranging from VND 9000 to VND 45,000 for 500 g of water spinach, encompassing the range of actual market prices, thereby enabling us to identify willingness to pay for the examined attributes [41].

A pilot choice experiment without using priors (n = 25) was designed using JMP^®^14 software (SAS Institute). Based on pilot results, Multinomial Logit (MNL) modelling was used to generate MNL parameter estimates, which later served as Bayesian priors to create the final Bayesian D-efficient design [42]. A total of sixteen choice sets were used, which were divided into two blocks. Each respondent received only one block of eight randomly selected choice sets to reduce cognitive burdens and avoid order bias [43,44]. Before the choice experiment, a cheap talk script was provided and the meaning of each attribute and its attribute levels was explained to minimise hypothetical bias [45]. In case respondents chose the opt-out, they were asked about their reasons for having done so.

### 3.3. Data Analysis

#### 3.3.1. Newly Constructed Variables

The multi-item variables, namely trust, familiarity, purchase habit and purchase intention, were verified with Cronbach’s alpha coefficients to ensure internal consistency and reliability. Items were merged into construct scores if considered sufficiently reliable, i.e., if Cronbach’s alpha coefficients were higher than 0.6 [46]. Finally, descriptive statistics were performed on socio-economic and other individual characteristics after having constructed individual construct scores.

#### 3.3.2. Econometric Models

Generalised multinomial logit (GMNL) models can take into account taste and scale heterogeneity among respondents, unlike other choice models such as the multinomial logit (MNL) and random parameter logit (RPL) models [47]. Taste heterogeneity indicates differences in valuation among consumers. Scale heterogeneity accounts for the randomness in the decision-making process. For example, participants might choose an alternative based on just one attribute instead of considering all attributes (lexicographic behaviour). Alternatively, participants might randomly choose an option as none of the desired product attributes is present in the choice set [48].

Fiebig et al. [47] developed a Generalised Multinomial Logit (GMNL) model to analyse taste and scale heterogeneity. In GMNL models, a vector of utility weights ($\beta_{n})$ of the n-specific participant can be described as follows (Equation (1)):

(1) $$\beta_{n}=\sigma_{n}\beta +\left[\gamma +\left(1-\gamma \right)\;\sigma_{n}\right]\eta_{n}$$

where $\sigma_{n}$ is the individual specific scale of the idiosyncratic error term and γ is a scalar parameter that controls how the variance of residual taste heterogeneity $\eta_{n}$ varies with scales. The empirical model specification for the GMNL model can be stated as follows (Equation (2)), given four attributes and the opt-out:

(2) $$\begin{array}{cc} U_{njt}=\sigma_{n}\beta_{Price}Price_{njt}+\;\beta_{No\;choice}No\;choice_{njt} & \\ +\left(\sigma_{n}\beta_{VietGAP,n}+\left[\gamma +\left(1-\gamma \right)\sigma_{n}\right]\eta_{VietGAP,n}\right)VietGAP_{njt} & \\ +\left(\sigma_{n}\beta_{Organic,n}+\left[\gamma +\left(1-\gamma \right)\sigma_{n}\right]\eta_{Organic,n}\right)Organic_{njt} & \\ +\left(\sigma_{n}\beta_{Branding,n}+\left[\gamma +\left(1-\gamma \right)\sigma_{n}\right]\eta_{Branding,n}\right)Branding_{njt} & \\ +\left(\sigma_{n}\beta_{Traceability,n}+\left[\gamma +\left(1-\gamma \right)\sigma_{n}\right]\eta_{Tracebility,n}\right)Traceability_{njt}+\varepsilon_{njt} \end{array}$$

at the beginning of this study, uncorrelated and correlated MNL, RPL and GMNL models were estimated in RStudio (version 1.2.5042), using the *mlogit* package for data formatting and the *gmnl* package for model estimation [49]. First, all econometric models were estimated with 500 Halton draws. Then, Akaike’s information criteria (AIC), log-likelihoods and the Bayesian information criteria (BIC) were assessed as indicators for the goodness-of-fit and model selection. Among the examined models, the goodness-of-fit indicators showed that the correlated GMNL model fitted best to the dataset. To simplify the result section, only the results of the correlated GMNL model are presented.

Effect coding was employed for utility estimation (Table 1). Unlike dummy coding, effect coding has the advantage of allowing the estimation of all attribute levels and allows for uncorrelated estimates with the intercepts [50].

#### 3.3.3. Willingness-to-Pay (WTP) Calculation

The WTP mean values of quality attributes were calculated based on the coefficient of the Price attribute as given by Equation (3):

(3) $$WTP_{k}=\frac{-\left(\beta_{k}-\beta_{k0}\right)\;}{\beta_{Price}}$$

where $\beta_{k}$ is the estimated coefficient for the examined attribute levels,$\;\beta_{k0}$ is the base level of $\beta_{k}$, and $\beta_{Price}$ is the estimated Price coefficient.

Effect coding enables the computation of the WTP for all attribute levels, including for the base levels of the attributes. The absence of binary attributes such as “No Branding” and “No Traceability” is associated with those attributes’ negative coefficients. Consequently, the utility difference with and without the presence of a binary attribute is the coefficient of that attribute multiplied by two. The WTP to switch from one level of an attribute to another is the difference in the corresponding coefficients [51]. The WTP to switch from a base level (“No Certification”, “No Branding”, and “No Traceability”) to another level is the price premium that a consumer is willing to pay for better-guaranteed quality, as represented by a certification, brand or traceability system.

## 4. Results

### 4.1. Descriptive Analyses

Descriptive analyses (Table 2) show that female consumers were dominant in this survey, which is in line with other food consumer surveys in Vietnam, indicating that women are predominately responsible for food shopping [22]. The sample’s age distribution is similar to that of the Vietnamese population [52]. About 70% of the respondents have obtained higher education, which is higher than the country population’s average education level.

### 4.2. Individual Specific Variables and Stratification

The newly constructed individual variables (Table 3), namely trust, familiarity, purchase habit and purchase intention, were found to be reliable based on their Cronbach’s alpha (all alpha’s > 0.98). Generally, respondents were slightly familiar with the examined logos, by which the private brand logo was the most familiar, while the organic logos were the least familiar logos (Table 3). No significant differences between consumer trust in VietGAP versus organic logos were found. By contrast, trust in the private brand was significantly lower than trust in VietGAP and organic logos. Respondents were found to have more knowledge about VietGAP certification than organic certifications. On average, they also reported occasional purchases of VietGAP and organic certified water spinach (four times out of ten, on average). The reported purchase frequency of VietGAP certified water spinach was also higher than that of organic certified water spinach. By contrast, there were no significant differences in purchase intention between VietGAP and organic certified water spinach.

On average, higher-income respondents were more familiar with organic certifications and the private brand than lower-income ones, while the inverse was found in the case of VietGAP logos (Table 4). Similarly, higher-educated respondents were more familiar with VietGAP, organic certifications and the private brand than lower-educated respondents. Lower-income and lower-educated respondents reported higher trust in VietGAP and the private brand, while their counterparts trusted organic logos more.

Higher-educated respondents were shown to have greater knowledge of VietGAP and organic certifications than lower-educated respondents. Furthermore, higher-educated respondents reported a higher frequency of purchase for both certifications than their counterparts. By contrast, their purchase intention was lower than lower-educated respondents.

When comparing lower- and higher-income respondents, higher-income respondents were found to have greater knowledge of organic certification. Similarly, higher-income respondents reported a higher purchase frequency and intention for organic certification than lower-income ones. By contrast, no significant differences were found in the knowledge of VietGAP certification between both income groups. On the other hand, lower-income respondents reported a greater purchase frequency and a higher purchase intention than higher-income ones for VietGAP certification.

### 4.3. Discrete Choice Models

After having assessed Akaike’s information criteria (AIC), the log-likelihoods and the Bayesian information criteria (BIC), the correlated GMNL model showed the best fit to the data; therefore, given the GMNL model’s mentioned advantages and its greater goodness-of-fit than correlated and uncorrelated MNL and RPL models, we only present the results of the GMNL model (Table 5).

All examined attributes, including the opt-out but excluding price, generated positive utilities (Table 5). Significant standard deviations of coefficients reveal taste heterogeneity among consumers, while significant τ indicates the presence of scale heterogeneity. Familiarity and knowledge were not associated with consumers’ valuation of examined attributes. While trust was found to be positively associated with consumers’ valuation of VietGAP-certified products, no association was found between trust and the valuation of organic certification and private brand. Higher income increased consumers’ valuation of EU and USDA organic logos but decreased the valuation of VietGAP logos while no association with private brand valuation was found. Similarly, higher-income respondents reported a higher purchase frequency and intention for organic certification than lower-income ones. By contrast, higher education levels reduced consumers’ preference for organic logos but increased the valuation of VietGAP and the private brand logo. Among the studied variables, none were associated with consumers’ valuation of traceability.

**Figure 2:**
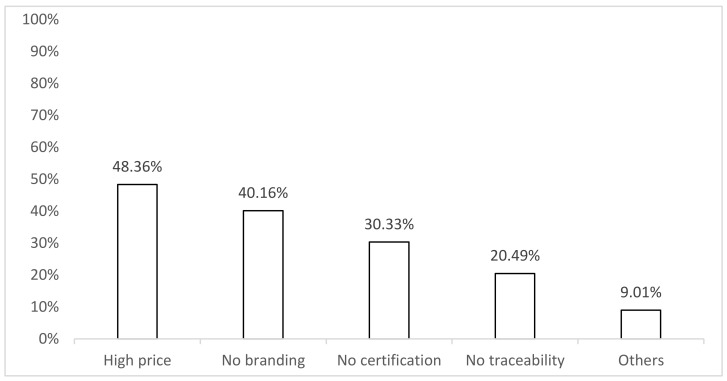
Reasons for choosing opt-out options ^a^ (%, n = 122). ^a^ Consumers were asked why they chose opt-out options (if applicable). Each consumer could choose more than one reason. The percentage of each reason = (number of times the reason was chosen / number of respondents choosing the opt-out option) × 100%.

Among the reasons for opting out, high prices were the most common (48%), followed by a lack of a private brand logo (40%) (Figure 2). Other reasons reported for opting out were disliking water spinach or self-cultivation.

### 4.4. Willingness to Pay

All the examined attributes were found to have a positive willingness to pay (Table 6). VietGAP certification was found to receive the lowest valuation amongst the assessed attributes, only valued at VND 8250 (€0.30). Nonetheless, consumers were willing to pay up to VND 57,150 (€2.05) to switch from a non-certified product to a VietGAP certified product (WTP VietGAP—WTP No Certification). By contrast, certified organic products were found to receive the highest valuation by consumers, reflected in the implicit price for EU and USDA organic water spinach reaching VND 40,650 (€1.46). This implies that consumers were willing to pay a price premium of VND 89,550 (€3.21) to switch from a conventional product without EU and USDA organic certifications to a certified organic one (WTP EU and USDA organic—WTP No Certification). Consumers valued the private brand logo at VND 25,420 (€0.91), and they were willing to pay a price premium of VND 50,840 (€1.82) to switch from a non-branded product to a branded one. Consumers also showed a positive valuation for the traceability attribute as they were willing to pay a premium of VND 33,070 (€1.18) to switch to water spinach with a traceability QR code.

## 5. Discussion

Our study found that the sampled Vietnamese consumers valued all examined attributes, including organic and VietGAP certifications, private branding and traceability. These findings align with previous studies on certifications, traceability and branding in Vietnam [21,31]. This study found no association between consumer preferences and familiarity with nor objective knowledge of the certification schemes. By contrast, trust, education and income were significantly associated with the consumer valuation of several attributes.

Familiarity with the logos of VietGAP, organic or the examined private brand was not significantly associated with consumers’ preference for the corresponding attribute levels. This finding is similar to an experiment on Fairtrade logos, where the official logo they were familiar with did not increase consumers’ preference compared to fictional Fairtrade logos [55]. Furthermore, when encountering a familiar certification logo, consumers might less deliberatively consider the value of the concerned certification, thereby not leading to an increased preference for the attribute [55].

Similarly, knowledge of VietGAP and organic certifications did not result in consumers’ preference for water spinach with the related certification logos. This finding is in line with Pieniak et al. [33], who indicated that objective knowledge (or actual knowledge) was only indirectly associated with organic vegetable consumption through improving subjective knowledge (perceived self-competence) and general attitude towards organic products.

By contrast, trust was shown to be associated with consumers’ valuation of VietGAP certified water spinach. Due to recent scandals regarding VietGAP certification, consumers might have less confidence in VietGAP certified production accountability [31]. VietGAP producers have been falsely labelling VietGAP logos for the mixture of conventional and VietGAP vegetables, fabricating logos, or using VietGAP logos without valid certifications [56]. Additionally, as there is currently no unified VietGAP logo [22], consumers might have difficulty recognising credible VietGAP logos, let alone identifying the counterfeit ones. Hence, the Vietnamese government could consider adopting a robust regulatory system to guarantee uniformity and transparency of certification practices as an opportunity. A unified and credible logo of VietGAP could be issued to help consumers make more informed purchase decisions considering food safety. Meanwhile, trust was not significantly associated with the valuation of EU and USDA organic certifications in this study. Thus, more studies should be conducted to investigate further variables affecting consumer valuation of international organic certifications in emerging countries.

Income levels were significantly associated with consumers’ preference for certified products. The high-income group reported preferring EU and USDA organic vegetables (Table 5). Due to relatively higher prices, organic products are currently more accessible to affluent consumers in Vietnam. Further, organic certifications were shown to be less familiar to consumers than other certifications such as VietGAP (Table 3). Hence, Vietnamese retailers and traders could consider employing marketing strategies to promote USDA and EU organic certified products, given their high price premium. By contrast, VietGAP certified products were more appealing to lower-income consumers (Table 4), possibly due to their lower market price.

Higher education levels were positively associated with consumers’ valuation of VietGAP logos, but negatively associated with the valuation of EU and USDA organic and brand logos (Table 5). The positive association between education and consumers’ valuation of VietGAP logos are in line with the findings of Zulfikar et al. [57], which indicated that high-educated consumers are willing to pay a price premium for GlobalGAP certified products. Notwithstanding that lower-educated consumers have less knowledge of organic certifications (Table 4), the results show that the lower the education level is, the higher the utility consumers expect to earn from the EU and USDA organic certifications (Table 5). This finding might imply that EU and USDA organic certification logos can attract Vietnamese consumers regardless of their knowledge of these certifications. Furthermore, as certification schemes are only slightly familiar to Vietnamese consumers (Table 3), a credible brand logo was still essential for consumers to make purchase decisions at the point of sale, especially for lower-educated consumers (Table 5). The study sample had a larger proportion of higher-educated respondents (Table 2); therefore, even though it is common that less-educated respondents are underrepresented compared to national statistics [58], care should be taken when extrapolating the results of this study with respect to the effect of familiarity with and knowledge of the examined certification schemes.

The WTP for the examined attributes offers important insights for marketing strategies and policy development. In this study, USDA and EU organic logos received a substantially higher valuation than other product attributes. Similarly, Chinese consumers expressed the highest WTP for the EU organic label compared to other organic certification schemes in Chinese markets [20]. International organic certifications could gain premium prices due to the increasing demand for imported high-quality products among Vietnamese consumers [38]. These results indicate the potential of price premia for international organic products, especially in supermarkets and other retail outlets. Hai et al. [8] suggested that the high price premium for organic products can foster organic farming development in the early stage. To maintain the momentum of organic farming development, the Vietnamese government and private sectors could implement effective market inspections for organic certifications to avoid food safety and quality scandals, as has been the case for VietGAP.

Given the price premium respondents reported to be willing to pay for branded products (Table 5) and the high opt-out rate for non-branded products in our study (Figure 2), branding evidently plays a critical role in food purchase decisions of water spinach in Vietnam and possibly also when purchasing other vegetables. Thus, Vietnamese food producers could consider investing more in food safety and quality management and build their brand images thereupon.

This study indicates the potential benefits of improving food traceability as Vietnamese consumers were willing to pay a significant price premium for such attributes. Hence, food chain actors and stakeholders could consider implementing traceability schemes to provide sufficient information and assure food safety and quality. Furthermore, Dang et al. [31] described the promising market segment for certified traceable food in Vietnam as consumers who, on average, are less than 35 years old, married, well-educated and above middle income. By contrast, our study did not find any significant association between consumers’ income, education and age and their preference for QR traceable products. Thus, more studies could contribute to a better understanding of consumers’ perception of and preferences for food traceability characteristics in Vietnam and other emerging countries.

Regarding limitations, our study was limited to two attribute levels of traceability and branding to avoid excessive cognitive burdens. Nonetheless, future studies could investigate additional levels of traceability attributes. Such studies could facilitate the market-driven establishment of traceability schemes as their implementation tends to be costly, and their success depends largely on consumers’ preferences [59]. Moreover, Larceneux et al. [60] indicated that organic labelling’s marginal effect on perceived product quality depended on brand equity. According to their study, the higher brand equity is, the less effective organic labels are and vice versa. In a similar vein, Van Loo et al. [61] indicated that half of their surveyed Belgian consumer sample valued private-label branding—similar to the ‘Coop Food’ brand concept used in this study—and organic production for eggs. Their study underscored the larger potential of food quality labelling and certification for private label (i.e., retail or store) branded products compared to national (i.e., manufacturer or producer) branded food products in a developed country context. Hence, future studies could consider multiple brands or brand concepts (e.g., private label versus national branding) to evaluate existing brand equity effects.

## 6. Conclusions

This study sheds light on consumers’ preferences for food labelling attributes of water spinach in Vietnam, where food safety issues and weak food law enforcement are prevalent, as in many other emerging countries. This study shows that trust is significantly associated with consumers’ valuation for VietGAP certified products. Thus, VietGAP certifiers are recommended to consider imposing more stringent regulations and regular inspections to regain consumers’ confidence and increase VietGAP certified products’ mark-up. Meanwhile, international organic certification may remain out of reach for lower-income consumers due to the higher price. Nonetheless, international organic certifications are highly valued by higher-income consumers. Food corporations and retailers can consider investing more in building and maintaining a favourable brand image, as brand logos are the most recognisable information and highly important for consumers to make food purchases at the point of sale in Vietnam. Another promising practice for food chain actors to gain consumers’ trust in food systems and products could be implementing traceability schemes. Even though communication on traceability is relatively new to emerging markets, our findings indicated significant consumer preference for traceability schemes. Lastly, the presence of taste and scale heterogeneity in consumers’ valuation of labelling schemes in our study emphasises the importance of further investigating market segmentation and consumer behaviour concerning food labelling schemes in Vietnam and other emerging consumers. Such studies would pave the way to develop more market-oriented agricultural products with better food safety and quality in line with consumer preferences in emerging countries.

## Figures and Tables

**Table 1:** Effect coding for attributes and their levels in the discrete choice experiment.

Attributes	Level	Effect Coding	
Price (1000 VND/500 g)	9.0; 16.2; 20.4; 30.6; 37.8; 45.0	Continuous variable	
Certification	VietGAP	1	0
	EU and USDA organic	0	1
	Conventional (No certification) *	−1	−1
Branding	Private brand (Coop Food)	1	
	No brand *	−1	
Traceability	QR code	1	
	No QR code *	−1	

* No certification, No brand, No QR code are the base levels.

**Table 2:** Socio-economic characteristics of the sample (n = 275).

Variables	Description	Sample (%)
Gender	0 = Female	92.73
	1 = Male	7.27
Age	20–29 years	16.73
	30–39 years	34.91
	40–49 years	25.45
	50–59 years	16.36
	>60 years	6.55
Monthly income	1 = less than 3,000,000 VND;	2.91
	2 = 3,000,001–6,000,000 VND;	8.00
	3 = 6,000,001–9,000,000 VND;	17.82
	4 = 9,000,001–12,000,000 VND;	20.36
	5 = 12,000,001–15,000,000 VND;	17.45
	6 = more than 15,000,000 VND	33.45
Education	1 = Primary school;	0.36
	2 = Secondary school;	5.82
	3 = High school;	18.18
	4 = College/University;	66.91
	5 = Postgraduate	8.73

**Table 3:** Descriptive statistics (mean scores and Standard Deviations (SD)) on individual specific variables (n = 275).

Variables	Mean (SD)
**Familiarity** (7-point Likert scales)	
VietGAP	5.31 ^b^ (1.38)
Organic	4.35 ^c^ (1.81)
Private brand	5.69 ^a^ (1.23)
**Trust** (7-point Likert scales)	
VietGAP	5.46 ^a^ (0.10)
Organic	5.32 ^a^ (1.31)
Private brand	5.05 ^b^ (1.17)
**Knowledge** * (4 true/false questions)	
VietGAP	3.26 ^d^ (1.26)
Organic	3.00 ^e^ (1.45)
**Purchase habit** (purchase frequency, times out of 10)	
VietGAP water spinach	4.27 ^d^ (2.80)
Organic water spinach	4.04 ^e^ (2.99)
**Purchase intention** (7-point Likert scales)	
VietGAP water spinach	5.22 ^d^ (1.15)
Organic water spinach	5.37 ^d^ (1.36)

^a, b, c^ indicate statistically significant differences in means of the variables (familiarity, trust) of labelling attributes based on ANOVA One-way tests and Bonferroni post hoc comparison (if applicable) at *p* < 0.05. ^d, e^ indicate statistically significant differences in means of the variables (knowledge, purchase habit and purchase intention) of certified products, based on Welch Two Sample t-tests at *p* < 0.05. * Four questions for knowledge assessment were based on the European Council Regulation (EC) No. 834/2007 for organic certifications and the general principles of VietGAP cultivation [53]. The composite knowledge score ranges from 0 (all answers wrong) to 4 (all answers correct).

**Table 4:** Association between respondents’ income and education level with other individual variables (n = 275).

	Income		Education	
	Lower-Income ^1^ (n = 79)	Higher-Income ^1^ (n = 196)	Lower-Educated ^2^ (n = 67)	Higher-Educated ^2^ (n = 208)
**Familiarity**				
VietGAP	5.48 ^a^ (1.28)	5.24 ^b^ (1.41)	5.02 ^b^ (1.55)	5.40 ^a^ (1.31)
Organic	4.23 ^b^ (1.77)	4.40 ^a^ (1.82)	4.16 ^b^ (1.72)	4.41 ^a^ (1.83)
Private brand	5.48 ^b^ (1.30)	5.77 ^a^ (1.18)	5.21 ^b^ (1.49)	5.84 ^a^ (1.08)
**Trust**				
VietGAP	5.67 ^a^ (0.69)	5.38 ^b^ (1.09)	5.51 ^a^ (0.92)	5.45 ^b^ (1.02)
Organic	5.14 ^b^ (1.16)	5.39 ^a^ (1.36)	5.18 ^b^ (1.21)	5.36 ^a^ (1.34)
Private brand	5.30 ^a^ (0.97)	4.95 ^b^ (1.23)	5.18 ^a^ (1.13)	5.01 ^b^ (1.18)
**Knowledge**				
VietGAP	3.26 ^a^ (1.29)	3.26 ^a^ (1.26)	2.91 ^b^ (1.52)	3.37 ^a^ (1.15)
Organic	2.95 ^b^ (1.39)	3.02 ^a^ (1.48)	2.84 ^b^ (1.47)	3.05 ^a^ (1.44)
**Purchase habit**				
VietGAP water spinach	4.51 ^a^ (2.56)	4.17 ^b^ (2.88)	4.61 ^b^ (2.53)	5.02 ^a^ (3.28)
Organic water spinach	3.41 ^b^ (2.73)	4.30 ^a^ (3.06)	3.85 ^b^ (2.72)	4.86 ^a^ (3.33)
**Purchase intention**				
VietGAP water spinach	5.44 ^a^ (0.98)	5.13 ^b^ (1.20)	5.46 ^a^ (0.87)	5.14 ^b^ (1.22)
Organic water spinach	5.22 ^b^ (1.34)	5.44 ^a^ (1.36)	5.48 ^a^ (1.18)	5.34 ^b^ (1.41)

^1^ Cut-off point at 9 million Vietnam dongs (VND) per month, ≤9 million VND was categorised as lower-income, >9 million VND as higher-income. The median salary of experienced staff in Ho Chi Minh City was 10 million VND in 2019 [54]. ^2^ Cut-off point at high school education, ≤ high school education was categorised as lower-educated, >high school education as higher-educated. ^a, b^ Superscripts indicate statistically significant differences in means between income or between education groups based on Welch Two Sample *t*-test, *p* < 0.05.

**Table 5:** Results of the correlated GMNL model.

	Estimate	*p*-Value	Significance Level
**Attribute means**			
Price	−0.06	<0.001	***
No choice	0.29	<0.001	***
VietGAP	0.46	0.020	*
EU and USDA organic	2.28	<0.001	***
Branding	1.43	<0.001	***
Traceability	0.93	<0.001	***
**Attribute standard deviation**			
VietGAP	−1.74	<0.001	***
EU and USDA organic	2.56	<0.001	***
Traceability	0.74	<0.001	***
τ	0.83	<0.001	***
γ	−0.30	0.004	**
**Incorporated individual-specific variables (Z)**			
***VietGAP x Z***			
Familiarity	0.34	0.304	
Trust	2.43	<0.001	***
Knowledge	−0.28	0.429	
Income	−0.97	0.037	*
Education	1.19	0.007	**
***EU and USDA organic x Z***			
Familiarity	−0.19	0.597	
Trust	0.58	0.369	
Knowledge	0.578	0.209	
Income	2.35	0.002	**
Education	−1.88	0.007	**
***Branding x Z***			
Familiarity	0.37	0.413	
Trust	0.82	0.064	
Income	−0.08	0.749	
Education	−0.78	0.007	**
***Traceability x Z***			
Income	−0.05	0.813	
Education	−0.44	0.057	
***Base level means***			
No certification	−3.59		
No brand	−1.88		
No traceability	−1.09		
***Goodness-of-fit***			
Observations ^a^	2200		
Log-likelihood	−1599		
AIC	3267		
BIC	3460		

***, **, * indicate significance at 0.1%, 1% and 5% level, respectively. ^a^ Refers to the number of observations = number of respondents (275) × number of the choice sets per respondent (8). τ is the parameter that captures scale heterogeneity. γ is the scalar parameter that controls how the variance of residual taste heterogeneity $\eta_{n}$ varies with scales.

**Table 6:** Implicit prices for water spinach attribute levels (in thousand Vietnamese dongs, VND) ^a^.

	Mean	Standard Error	95% Confidence Interval	*p*-Value
**VietGAP**	8.25	3.54	[1.32;15.19]	0.019
**EU and USDA organic**	40.65	6.69	[27.54;53.76]	<0.001
**Branding**	25.42	4.72	[16.17;34.66]	<0.001
**Traceability**	16.53	2.83	[10.99;22.08]	<0.001

^a^ Unit: thousand Vietnamese dongs (VND) per 500 g of water spinach. WTP Base levels: WTP No Certification = −48.90; WTP No brand = −25.42; WTP No Traceability = −16.53. In February 2021, 1 EUR = 27,900 VND.

## Data Availability

Dataset is available upon request.

## Appendix A

**Table A1:** Description of individual specific variables.

Variable	Description			Scale	Sources
Familiarity with VietGAP, Organic, and Coop-Food logo	How familiar are you with the following logos?			Seven-point Likert scale: From *1-Extremely unfamiliar* to *7-Extremely familiar*	[22]
	VietGAP	Organic	Coop-Food		

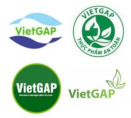
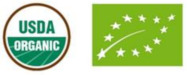
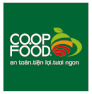
		
Trust in VietGAP certification	To what extent, you agree with the following statement: (1) I trust VietGAP labels; (2) VietGAP labels are certified; (3) VietGAP labels are credible; (4) VietGAP labels are a guarantee for the quality and safety of the product; (5) Certification bodies issued VietGAP labels after a careful assessment; (6) Certification bodies periodically inspect the VietGAP production process.			Seven-point Likert scale: From *1-Extremely disagree* to *7-Extremely agree*	[62,63,64]
Trust in Organic certification	To what extent, you agree with the following statement: (1) I trust Organic labels; (2) Organic labels are certified; (3) Organic labels are credible; (4) Organic labels are a guarantee for the quality and safety of the product; (5) Certification bodies issued Organic labels after a careful assessment; (6) Certification bodies periodically inspect the Organic production process.				
Trust in Coop-Food brand logo	To what extent, you agree with the following statement: (1) I trust this private brand label; (2) This private brand label is certified; (3) This private brand label is credible; (4) This private brand is a guarantee for the quality and safety of the product.				
Knowledge of VietGAP certification	Which statement below is true: (1) VietGAP farmers are trained and monitored by experts; (2) In VietGAP, heavy metal levels of soil are assessed to meet the allowed standard; (3) In VietGAP, irrigated water is qualified as domestic water for human consumption; (4) In VietGAP, the data of harvest and processing are recorded and traceable.			Correct = 1; Incorrect/Do not know = 0	[53]
Knowledge of Organic certification	Which statement below is true: (1) Organic farmers may use synthetic pesticides; (2) Organic farmers may use synthetic fertilisers; (3) Organic farmers may use genetically modified seeds; (4) Organic farmers may use preservatives to prolong vegetable shelf-life				[65]
Purchase habit for VietGAP/Organic water spinach	Out of 10 times that you buy water spinach, how often do you choose one with a VietGAP labelled product?			Ten-point interval scale: From *1* to *10*	-
	Out of 10 times that you buy water spinach, how often do you choose one with an Organically labelled product?				
Purchase intention for VietGAP/Organic water spinach	To which extent, you agree with these statements: (1) I expect to eat VietGAP water spinach in the coming 4 weeks; (2) I plan to eat VietGAP water spinach in the coming 4 weeks; (3) I desire to eat VietGAP water spinach in the coming 4 weeks			Seven-point Likert: From *1-Extremely disagree* to *7-Extremely agree*	[22]
	To which extent, you agree with these statements: (1) I expect to eat Organic water spinach in the coming 4 weeks; (2) I plan to eat Organic water spinach in the coming 4 weeks; (3) I desire to eat Organic water spinach in the coming 4 weeks.				
